# Segmentectomy Versus Lobectomy in Patients with Stage IA Lung Adenocarcinoma: Long-Term Survival in a Propensity Score-Matched Cohort

**DOI:** 10.3390/cancers18081202

**Published:** 2026-04-09

**Authors:** Zhangfeng Huang, Tenglong Luo, Zuhan Geng, Qi Gao, Yongde Liao

**Affiliations:** 1 Department of Thoracic Surgery, Union Hospital, Tongji Medical College, Huazhong University of Science and Technology, Wuhan 430022, China; 2 Department of Plastic Surgery, Union Hospital, Tongji Medical College, Huazhong University of Science and Technology, Wuhan 430022, China; 3 Department of Nephrology, Wuhan Fourth Hospital, Wuhan Clinical Research Center for Metabolic Chronic Kidney Disease, Wuhan 430030, China

**Keywords:** lung adenocarcinoma, early stage, segmentectomy, lobectomy, cohort study

## Abstract

**Background**: Whether there are differences in clinical outcomes between segmentectomy and lobectomy in patients with early-stage lung adenocarcinoma (LUAD) remains uncertain. This study aimed to compare all-cause mortality and lung cancer-specific mortality in patients with lung tumors ≤ 20 mm undergoing these two procedures. **Methods**: Patients with stage IA lung adenocarcinoma (≤20 mm) who underwent segmentectomy or lobectomy were identified from the SEER database (2008–2022). Propensity score matching (PSM) was applied to balance baseline characteristics. Kaplan–Meier curves depicted overall survival and lung cancer-specific survival. Multivariable Cox proportional hazards models were used to evaluate associations between surgical procedures and mortality, reporting hazard ratios (HRs) with 95% confidence intervals (CIs). **Results**: Among 9641 patients, 1065 (11.0%) underwent segmentectomy. After 1:1 PSM, 2028 patients had well-balanced covariates. The median follow-up was 43.0 months. In the lobectomy group, 158 all-cause deaths (35.1 per 1000 person-years) and 66 lung cancer-specific deaths (14.7 per 1000 person-years) occurred, compared with 176 and 80 events in the segmentectomy group (39.9 and 18.1 per 1000 person-years, respectively). Multivariable Cox regression demonstrated that segmentectomy, compared with lobectomy, was not associated with a higher risk of all-cause mortality (adjusted HR [aHR], 1.07, 95% CI 0.86–1.34) or lung cancer-specific mortality (aHR, 1.18, 95% CI 0.84–1.64). The results were consistent across tumor differentiation subgroups. **Conclusions**: Among patients with early-stage (≤20 mm) LUAD, segmentectomy was not associated with an increased risk of all-cause or lung cancer-specific mortality compared with lobectomy. Further studies with larger sample sizes are warranted to validate these findings.

## Simple Summary

Patients with stage IA lung adenocarcinoma ≤ 20 mm who underwent segmentectomy or lobectomy were identified from the SEER database (2008–2022). After propensity score matching, survival outcomes were compared using Kaplan–Meier analysis and multivariable Cox regression. We did not detect a significant difference in all-cause or cancer-specific mortality between the two surgical approaches.

## 1. Introduction

According to global cancer statistics, lung cancer remains the leading cause of cancer-related mortality worldwide, with an estimated 2.48 million new cases and 1.82 million deaths reported in 2022 [1]. Among non-small cell lung cancer (NSCLC), lung adenocarcinoma (LUAD) is the predominant histological subtype [2,3,4]. In recent years, the widespread implementation of low-dose computed tomography screening has substantially increased the detection of small (≤20 mm), early-stage LUAD, many of which exhibit indolent growth patterns [5,6,7,8]. Given their favorable prognosis, the necessity for extensive surgical resection has been increasingly questioned, leading to renewed interest in lung-preserving approaches such as segmentectomy, which aim to balance oncologic efficacy with preservation of pulmonary function [9,10].

Despite these advances in early detection, important clinical challenges remain. Although patients with early-stage LUAD generally demonstrate favorable survival outcomes, a proportion still experience recurrence following surgical resection [11,12]. Moreover, LUAD is a biologically heterogeneous disease, with variations in tumor size, radiological features, histological subtypes, and molecular characteristics that may influence tumor behavior and clinical outcomes [11,12,13]. These features are often associated with relatively indolent tumor behavior and lower invasive potential, providing a biological rationale for considering lung-preserving approaches such as segmentectomy.

Several randomized controlled trials, including JCOG0802/WJOG4607L and CALGB 140503 (Japan Clinical Oncology Group Trial 0802, JCOG0802; West Japan Oncology Group Trial 4607L, WJOG4607L; and Cancer and Leukemia Group B Trial 140503, CALGB 140503), have compared segmentectomy with lobectomy in patients with early-stage NSCLC [14,15,16,17]. These studies have shown that segmentectomy can achieve survival outcomes that are not inferior to those of lobectomy in selected patients. However, important uncertainties remain. First, these trials included heterogeneous patient populations and were not specifically designed to evaluate all clinically relevant subgroups. Second, updated long-term results from the JCOG0802 trial suggest that, although segmentectomy is not inferior to lobectomy in terms of overall survival (OS), it may not confer a clear survival advantage and is associated with a higher rate of local recurrence [18]. These findings highlight a potential trade-off between local control and parenchymal preservation, underscoring the need for further evidence to guide surgical decision-making.

Given the distinct clinical and molecular characteristics of LUAD, it is plausible that the balance between oncologic control and parenchymal preservation may vary across patient subgroups [19]. However, real-world evidence evaluating long-term outcomes of segmentectomy versus lobectomy in patients with small (≤20 mm), early-stage LUAD remains limited. Therefore, population-based analyses focusing on this population are needed to address this gap.

We hypothesized that long-term outcomes would be comparable between segmentectomy and lobectomy for patients with stage IA lung adenocarcinoma. Therefore, this study aimed to conduct a population-based cohort analysis using the Surveillance, Epidemiology, and End Results (SEER) database to compare all-cause and lung cancer-specific mortality between segmentectomy and lobectomy in patients with lung adenocarcinoma ≤ 20 mm.

## 2. Materials and Methods

### 2.1. Study Design, Population, and Data Source

This retrospective population-based cohort study was conducted using data from the SEER Program (https://seer.cancer.gov/, accessed on 31 December 2025). The SEER program represents a population-based cancer registry encompassing approximately 28% of all incident cancer cases in the United States and provides high-quality, standardized information on patient demographics, tumor characteristics, treatment patterns, and survival outcomes. A detailed description of the study design and database information has been previously reported. The specific inclusion and exclusion criteria applied in this study are summarized in Figure S1.

A total of 311,129 patients diagnosed with lung adenocarcinoma between 2008 and 2022 were identified from the SEER database. The exclusion criteria were as follows: (1) more than one primary tumor (*N* = 105,568); (2) non-stage IA (*N* = 175,857); (3) absence of surgical treatment (*N* = 8808); (4) receipt of surgical procedures other than lobectomy or segmentectomy (*N* = 4211); (5) receipt of neoadjuvant therapy, including chemotherapy, radiotherapy, or systemic therapy (*N* = 72); (6) unknown tumor laterality or unknown number of dissected lymph nodes (*N* = 1360); and (7) tumor size > 20 mm (*N* = 5612). Ultimately, 9641 patients with lung adenocarcinoma ≤ 20 mm who underwent segmentectomy or lobectomy were included in the final analysis (Figure S1).

This study was determined to be Non-Human Subjects Research by the Institutional Review Board (IRB) at Union Hospital, Tongji Medical College, Huazhong University of Science and Technology because it utilized de-identified, publicly available data from the SEER database. Consequently, formal IRB approval and individual informed consent were not required. All study procedures were conducted in accordance with the ethical standards of the Declaration of Helsinki.

### 2.2. Surgical Modalities

The primary exposure was the type of surgical procedure, categorized as segmentectomy or lobectomy. Surgical modalities were defined according to SEER database codes as segmentectomy (code: 21, or 22), and lobectomy (codes 30, or 33).

In this study, segmentectomy was defined as an anatomic segmentectomy. To ensure a homogeneous comparison, patients who underwent wedge resection (non-anatomic resection) were excluded, as this procedure differs from anatomic segmentectomy in both surgical technique and oncological rationale.

### 2.3. Primary Outcomes

The index date was defined as the date of first diagnosis of LUAD recorded in the SEER database. The primary outcomes were all-cause mortality and lung cancer-specific mortality. All-cause mortality was defined as death from any cause during the follow-up. Cause of death was obtained from the SEER variable “SEER cause-specific death classification”, which is derived from death certificate information coded using the International Classification of Diseases (ICD) and processed through SEER’s cause-specific death classification algorithm to reduce misclassification. Death status and follow-up were obtained from SEER vital status records, which are based on linkages to state vital statistics and the National Death Index. Follow-up in SEER is registry-based and passive. No additional external validation beyond SEER-provided linkages was performed. Lung cancer-specific mortality was defined as death attributable to lung cancer based on the SEER cause-specific death classification (coded as “Dead [attributable to this cancer diagnosis]”), while deaths due to other causes were treated as censored events.

Patients were followed from the index date until the occurrence of all-cause mortality or lung cancer-specific mortality, censoring, or the maximum follow-up time available in the SEER database (31 December 2022), whichever came first.

### 2.4. Covariates and Other Definitions

Covariates in this study were categorized into three groups: sociodemographic factors, tumor-related characteristics, and treatment-related variables. Sociodemographic factors included age, sex (female, or male), race/ethnicity (White, Black, or others), year of lung adenocarcinoma diagnosis (2008–2010, 2011–2015, 2016–2020, or 2021–2022), marital status (married, divorced, single, widowed, or others), and household income (<50 k, 50–<75 k, 75–<100 k, or >100 k). Tumor-related characteristics included tumor size, tumor laterality (left or right), tumor site (lower, middle, or upper), differentiation grade (poorly, moderately, or well differentiated), T stage (IA1 or IA2), and histological subtypes (lepidic, acinar, or others). Treatment-related variables included the number of lymph node stations examined (0, 1–3, or ≥4), and receipt of adjuvant radiotherapy (yes or no), chemotherapy (yes or no), or systemic therapy (yes or no).

Tumor size was obtained from the SEER variable “Tumor Size Over Time Recode (1988+)”, a harmonized variable that integrates tumor size information across coding systems and preferentially reflects pathologic tumor size when available, or clinical measurements based on imaging. Patients T stage was determined using pathology data according to the 9th edition of the Tumor node metastasis (TNM) classification. In addition, patients were additionally categorized into stage IA1 (≤10 mm) and IA2 (>10 to 20 mm) based on tumor size. Tumor grade was categorized according to SEER definitions as well differentiated (grade 1), moderately differentiated (grade 2), and poorly differentiated (grade 3), with cases of undifferentiated or unknown grade excluded. Histological subtypes were identified using the International Classification of Diseases for Oncology, Third Edition (ICD-O-3) morphology codes. Lung adenocarcinoma cases were defined using ICD-O-3 codes 8140, 8144, 8250–8254, 8230, 8260, 8265, 8310, 8333, 8480, 8490, and 8550–8551. Histological subtypes were further classified as lepidic (codes 8250, 8251, and 8252), acinar (codes 8550, 8551, and 8140), or others (codes 8144, 8230, 8253, 8254, 8260, 8265, 8310, 8333, 8480, and 8490).

### 2.5. Statistical Analysis

Continuous variables were presented as mean ± standard deviation (SD), and categorical variables were presented as frequencies (percentages). Differences between groups were compared using analysis of variance (ANOVA) for continuous variables and the chi-square test or Fisher’s exact test (if the expected value was <5) for categorical variables. Kaplan–Meier survival curves and the log-rank test were used to compare cumulative OS and lung cancer-specific survival (LCSS) between patients undergoing segmentectomy and lobectomy. The association between different surgical procedures (segmentectomy vs. lobectomy) and the primary outcomes was examined using Cox proportional hazards models, and the corresponding hazard ratios (HRs) with 95% confidence intervals (CIs) were reported. Three Cox models were constructed: Crude model; Model 1, adjusted for age, sex, and race/ethnicity; Model 2, further adjusted for year of diagnosis, marital status, household income, tumor size, laterality, tumor site, number of resected lymph node stations, number of resected lymph nodes, adjuvant chemotherapy, adjuvant radiotherapy, adjuvant systemic therapy, and differentiation grade. The proportional hazards assumption was evaluated using Schoenfeld residuals based on the cox.zph function in R.

In addition, to minimize baseline differences between the segmentectomy and lobectomy groups, propensity score matching (PSM) was performed using 1:1 nearest-neighbor matching within a caliper of 0.2× the SD of the propensity score (PS). A standardized mean difference (SMD) of <0.1 after matching was considered to indicate an acceptable balance in baseline characteristics between the two groups. PS were estimated using a multivariable logistic regression model that included the same covariates as Model 2, with the surgical procedure (segmentectomy vs. lobectomy) as the dependent variable. All subsequent analyses were conducted in the 1:1 PSM cohort [20].

We prespecified several subgroup analyses to explore potential effect modifiers of the association between surgical procedures (segmentectomy vs. lobectomy) and OS and LCSS. Predefined subgroup included age (≤65 vs. >65 years), sex (female vs. male), race/ethnicity (white and non-white), tumor size (≤10 vs. >10–20 mm), tumor laterality (left vs. right), the number of lymph nodes resected (median value = 7; ≤7 vs. >7) and histological subtypes (lepidic vs. acinar vs. others). Multivariable Cox models were fitted within each subgroup, and interaction terms were tested to evaluate statistical significance.

### 2.6. Sensitivity Analyses Methods

To further validate the robustness of our findings, several sensitivity analyses were conducted. First, deaths from causes other than lung cancer were treated as competing events for lung cancer-specific mortality, and the association between surgical procedure and lung cancer-specific mortality was re-evaluated using the Fine–Gray competing risk model. Second, the inverse probability of treatment weighting (IPTW) Cox model was applied to reassess the results, and weights were assigned as 1/PS for patients in the segmentectomy group and 1/(1 − PS) for those in the lobectomy group. Notably, the IPTW was performed based on the original pre-matching cohort (*N* = 9641), and the Fine–Gray competing risk analysis was conducted based on the primary 1:1 PSM cohort. Lastly, a more stringent propensity score matching criterion was used, in which the analysis was repeated using 1:1 nearest-neighbor matching within a caliper of 0.1× the SD of the propensity score.

All analyses were performed using R, Version 4.5.1 (https://www.r-project.org/, accessed on 31 January 2026), with two-tailed *p* values < 0.05 considered statistically significant.

## 3. Results

### 3.1. Baseline Characteristics of Study Population

A total of 9641 patients with stage IA lung adenocarcinoma who underwent segmentectomy or lobectomy were included in the study, of whom 1056 received segmentectomy and 8585 received lobectomy. After 1:1 PSM, 2082 patients (1041 in each group) were retained. Overall, baseline characteristics were well balanced between the two groups after matching (all SMD < 0.1, Table 1). The mean age of the cohort was 67.8 ± 8.9 years, and the majority were female (67.9%). The mean tumor size was 13.2 ± 4.3 mm, with stage IA2 comprising the majority (71.9%). Pathological differentiation was mainly moderate (75.0%).

Baseline characteristics of patients stratified by all-cause mortality before and after 1:1 PSM are shown in Table S1. In the matched cohort, patients who experienced all-cause mortality were older at baseline (70.9 vs. 67.1 years), had larger tumors (14.4 vs. 13.0 mm), and had a higher proportion of moderately to poorly differentiated tumors (91.6% vs. 87.6%).

### 3.2. Survival Analyses

**Figure 1:**
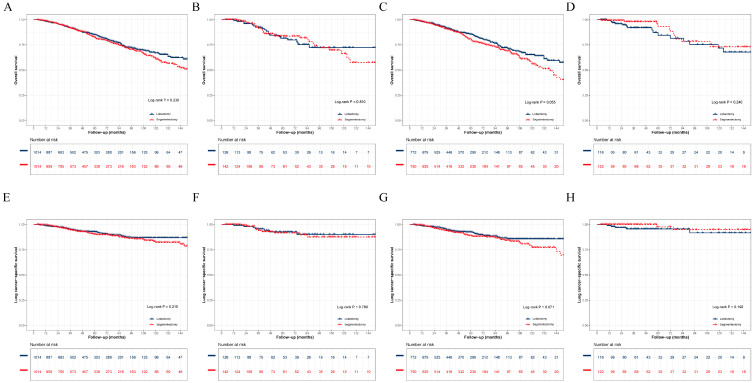
Overall survival or Lung Cancer-Specific Survival analysis with stage IA (≤20 mm) lung adenocarcinoma undergoing lobectomy or segmentectomy after 1:1 PSM. (**A**) OS for total patients; (**B**) OS in patients with poorly differentiation; (**C**) OS in patients with moderately differentiation; (**D**) OS in patients with well differentiation; (**E**) LCSS for total patients; (**F**) LCSS in patients with poorly differentiation; (**G**) LCSS in patients with moderately differentiation; (**H**) LCSS in patients with well differentiation. Abbreviations: OS, overall survival; PSM, propensity score matching; LCSS, lung cancer-specific survival.

In the matched cohort of 2028 patients, the median follow-up was 43.0 months (interquartile range, 19.0–76.0 months). During follow-up, 158 all-cause deaths (35.1 per 1000 person-years) and 66 lung cancer-specific deaths (14.7 per 1000 person-years) occurred in the lobectomy group, while 176 all-cause deaths (39.9 per 1000 person-years) and 80 lung cancer-specific deaths (18.1 per 1000 person-years) occurred in the segmentectomy group. Kaplan–Meier curves were used to compare OS and LCSS between segmentectomy and lobectomy in the overall cohort and across subgroups stratified by tumor differentiation (Figure 1). For OS, no significant differences were observed between the two surgical procedures in the overall population (Figure 1A) or in subgroups with poorly differentiated (Figure 1B), moderately differentiated (Figure 1C), and well-differentiated tumors (Figure 1D) (all log-rank *p* > 0.05). Similarly, for LCSS, comparable results were observed between segmentectomy and lobectomy in the overall cohort (Figure 1E) and across the corresponding subgroups (Figure 1F–H) (all log-rank *p* > 0.05).

The proportional hazards assumption was not violated (all *p* > 0.05, Table S2). Multivariable Cox regression analysis showed that patients undergoing segmentectomy had similar risks of all-cause mortality (adjusted HR [aHR]: 1.07; 95% CI: 0.86–1.34; Table 2) and lung cancer-specific mortality (aHR: 1.18; 95% CI: 0.84–1.64; Table 2) compared with those undergoing lobectomy.

Furthermore, multivariable Cox analyses did not find an increased risk of all-cause mortality for patients undergoing segmentectomy compared with lobectomy across differentiation subgroups (poorly: aHR 0.84; 95% CI: 0.46–1.54; moderately: aHR 1.18; 95% CI 0.93–1.52; well: aHR 0.60; 95% CI 0.28–1.28; Table 3). Similarly, segmentectomy was not associated with a higher risk of lung cancer-specific mortality in any differentiation subgroup (poorly: aHR 0.91; 95% CI: 0.34–2.47; moderately: aHR 1.31; 95% CI 0.91–1.89; well: aHR 0.28; 95% CI 0.05–1.46; Table 3). Notably, some subgroup estimates, particularly in the well-differentiated group for cancer-specific mortality, are based on a small number of events and are associated with wide confidence intervals. These estimates may be unstable and should be interpreted with caution.

**Figure 2:**
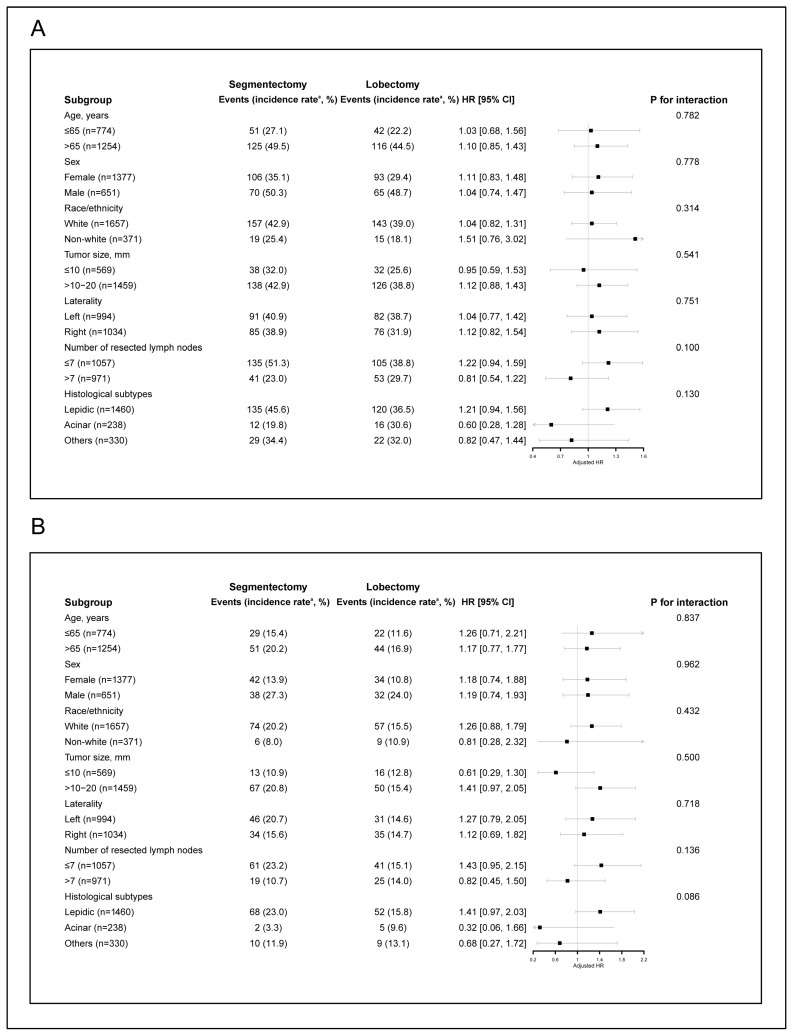
Forest plots of multivariable Cox proportional hazards regression model by subgroup analyses after 1:1 PSM for all-cause mortality (**A**) and lung cancer-specific mortality (**B**). ^a^ incidence rate: per 1000 person-years. Abbreviations: PSM, propensity score matching; HR, hazard ratio.

None of the variables, including age, sex, race/ethnicity, tumor size, tumor laterality, the number of resected lymph nodes, or histological subtypes, significantly modified the relationship between surgical procedure and either all-cause mortality or lung cancer-specific mortality (all *p* for interaction >0.05; Figure 2). These subgroup analyses were prespecified and interpreted cautiously given the potential for limited statistical power. In addition, exploratory analyses were conducted without formal adjustment for multiple comparisons; therefore, the interaction results should be interpreted with caution.

### 3.3. Sensitivity Analyses

The results of multiple sensitivity analyses were consistent with the primary findings. When deaths from non-lung cancer causes were treated as competing events, the multivariable Fine–Gray competing risk model did not suggest an increased mortality risk associated with segmentectomy (aHR 1.15; 95% CI 0.82–1.62; Table S3). After applying IPTW, the risks of all-cause mortality and lung cancer-specific mortality remained comparable between segmentectomy and lobectomy groups (all-cause mortality: aHR 1.14; 95% CI 0.94–1.39; lung cancer-specific mortality: aHR 1.20; 95% CI 0.91–1.59; Table S4). Finally, using a more stringent matching criterion, 1014 matched pairs were generated, and the results remained consistent with the main analysis (all-cause mortality: aHR 1.08; 95% CI 0.87–1.36; lung cancer-specific mortality: aHR 1.20; 95% CI 0.86–1.69; Table S5).

## 4. Discussion

In this population-based study, we found that among patients with stage IA lung adenocarcinoma (≤20 mm), segmentectomy was not associated with an increased risk of all-cause or lung cancer–specific mortality compared with lobectomy after adjustment for multiple confounding factors. Additionally, consistent findings were observed across several subgroup analyses and sensitive analyses.

Our findings are consistent with accumulating evidence suggesting that segmentectomy may achieve oncologic outcomes comparable to lobectomy for small peripheral LUAD. The JCOG0802/WJOG4607L randomized trial reported a significant OS advantage for segmentectomy over lobectomy in tumors ≤ 20 mm, despite a higher rate of local recurrence. However, a subsequent long-term update (2025), presented as a conference report rather than a fully peer-reviewed publication, suggested attenuation of this survival advantage, with no statistically significant difference observed at extended follow-up [18]. Collectively, these data suggest a potential trade-off between improved local control and preservation of lung parenchyma, rather than a definitive survival advantage of either approach. Notably, although segmentectomy was associated with a higher local recurrence rate in the randomized setting, our SEER-based analysis did not demonstrate an increased risk of either all-cause or lung cancer-specific mortality with segmentectomy. Several factors may explain this discrepancy. First, SEER does not capture recurrence outcomes, precluding direct comparison of local recurrence rates. Second, local recurrence does not necessarily lead to death, as effective salvage treatments may mitigate its impact on survival. Third, competing risks in early-stage populations may dilute differences in lung cancer–specific mortality. Additionally, differences in follow-up duration and residual confounding inherent to observational data, including unmeasured factors such as pulmonary function, smoking, surgical margins, and lymph node evaluation, may further contribute to this inconsistency. Therefore, a higher rate of local recurrence may not necessarily correspond to an increased risk of mortality. In this context, our findings–showing no detected difference in mortality between segmentectomy and lobectomy in patients with tumors ≤ 20 mm–are broadly consistent with both randomized and observational evidence. However, given the observational design and the potential for residual confounding, these results should be interpreted cautiously and should not be considered definitive evidence of equivalence.

Tumor differentiation is a well-established prognostic factor in LUAD, with poor differentiation consistently associated with worse survival outcomes. Previous studies have shown that poorly differentiated tumors are linked to a higher risk of recurrence and may warrant more aggressive treatment strategies [21,22,23]. However, in the present study, subgroup analyses based on tumor differentiation demonstrated no significant interaction between differentiation grade and surgical procedure. Notably, even among patients with poorly differentiated tumors, segmentectomy was not associated with an increased risk of lung cancer-specific mortality compared with lobectomy. These findings suggest no significant differences in oncologic outcomes between segmentectomy and lobectomy across different differentiation subgroups. Several subgroup estimates were associated with wide confidence intervals, particularly in strata with very low event counts (e.g., well-differentiated tumors for cancer-specific mortality), indicating limited statistical power and imprecision.

Lymph node evaluation is a key determinant of staging accuracy and survival. In addition to surgical resection, the extent and number of lymph node assessment play a pivotal role in determining oncologic adequacy during segmentectomy. Adequate retrieval and examination of hilar and mediastinal lymph nodes are essential to avoid understaging, ensure appropriate adjuvant treatment decisions, and accurately assess long-term prognosis. Notably, contemporary evidence indicates that comprehensive nodal evaluation can be reliably achieved during segmentectomy, regardless of the operative modality. Studies comparing robot-assisted (RATS), video-assisted thoracoscopic surgery (VATS), and open segmentectomy have reported similar nodal upstaging rates, suggesting that minimally invasive techniques do not compromise the thoroughness of lymphadenectomy [24,25]. There is a necessity for systematic lymph node dissection during segmentectomy, whether RATS, VATS, or open thoracotomy, and instead depend primarily on the adequacy of nodal evaluation. Even when the total number of retrieved lymph nodes is relatively low, standardized lymphadenectomy combined with propensity score-adjusted analyses has been shown to maintain accurate staging and robust oncologic outcomes. These findings provide additional evidence supporting the use of segmentectomy in the management of early-stage NSCLC [26,27,28,29]. In our study, subgroup analyses stratified by lymph node number (≤7 vs. >7 nodes) further confirmed that the survival effect of surgical type was not modified by the extent of lymph node evaluation. The absence of excess cancer-specific mortality in the segmentectomy cohort, even among tumors with poorer differentiation, suggests that, when adequate nodal assessment is performed, segmentectomy may not compromise staging accuracy or long-term survival.

This study has several limitations. First, as an observational study, it cannot establish a causal relationship between segmentectomy/lobectomy and long-term mortality. Second, although we adjusted for a range of sociodemographic factors, tumor-related characteristics, and treatment-related variables, several important clinical factors were not available in the SEER database, including smoking history, pulmonary function, chronic obstructive pulmonary disease, or usual interstitial pneumonia, surgeon expertise, margin distance, VATS/robotic approach, and detailed information on lymphadenectomy. These unmeasured factors may influence both treatment selection and survival outcomes. For example, smoking history is closely associated with lung cancer development and may also affect treatment response and prognosis [30]. Pulmonary function is a key determinant of surgical eligibility, while the extent and thoroughness of lymph node dissection directly affect the accuracy of pathological staging and subsequent treatment decisions [31]. In addition, surgeon experience may influence surgical quality and the risk of perioperative complications, thereby indirectly impacting survival outcomes [32]. As these potentially important confounders are not captured in the SEER database, residual confounding may persist even after multivariable adjustment.

In addition, information on comorbid conditions is limited in SEER. These factors may influence both surgical decision-making and long-term outcomes. For instance, patients with significant comorbidities may be preferentially selected for less extensive resection, which could bias effect estimates. The lack of detailed data on these factors further contributes to residual confounding, the direction and magnitude of which are difficult to quantify.

Finally, as this study was based on a U.S. population, the generalizability of our findings to other regions may be limited due to differences in healthcare systems, surgical practices, and patient characteristics. Previous studies have highlighted potential regional variations; for example, the findings of the JCOG0802 trial may not be directly generalizable to Western populations, whereas real-world analyses in the United States have reported comparable survival outcomes between segmentectomy and lobectomy [8,16,33]. Similarly, evidence from Asian populations suggests that minimally invasive segmentectomy can achieve comparable long-term oncologic outcomes [34]. Therefore, further validation in diverse populations and healthcare settings is warranted.

## 5. Conclusions

Our study did not detect a significant difference in long-term all-cause or lung cancer-specific mortality between segmentectomy and lobectomy among patients with stage IA lung adenocarcinoma (≤20 mm). Given the observational design and the limited sample size, these findings should be interpreted with caution. Further studies with larger sample sizes are warranted.

## Figures and Tables

**Table 1:** Baseline characteristics of patients undergoing lobectomy or segmentectomy before and after 1:1 propensity score matching.

		Before Propensity Score Matching				After Propensity Score Matching		
Characteristics	Total (*n* = 9641)	Lobectomy (*n* = 8585)	Segmentectomy (*n* = 1056)	SMD	Total (*n* = 2028)	Lobectomy (*n* = 1014)	Segmentectomy (*n* = 1014)	SMD
Sociodemographic factors								
Age, years	66.3 ± 9.1	66.1 ± 9.1	67.9 ± 9.1	0.195	67.8 ± 8.9	67.9 ± 8.6	67.7 ± 9.1	0.020
Female	6270 (65.0)	5553 (64.7)	717 (67.9)	0.068	1377 (67.9)	692 (68.2)	685 (67.6)	0.015
Race/ethnicity, *n* (%)				0.057				0.019
White	7773 (80.6)	6904 (80.4)	869 (82.3)		1657 (81.7)	826 (81.5)	831 (82.0)	
Black	799 (8.3)	725 (8.4)	74 (7.0)		147 (7.2)	73 (7.2)	74 (7.3)	
Others	1069 (11.1)	956 (11.1)	113 (10.7)		224 (11.0)	115 (11.3)	109 (10.7)	
Year at diagnosis, *n* (%)				0.328				0.032
2008–2010	1345 (14.0)	1247 (14.5)	98 (9.3)		183 (9.0)	88 (8.7)	95 (9.4)	
2011–2015	2630 (27.3)	2417 (28.2)	213 (20.2)		410 (20.2)	202 (19.9)	208 (20.5)	
2016–2020	4067 (42.2)	3596 (41.9)	471 (44.6)		902 (44.5)	453 (44.7)	449 (44.3)	
2021–2022	1599 (16.6)	1325 (15.4)	274 (25.9)		533 (26.3)	271 (26.7)	262 (25.8)	
Marital status, *n* (%)				0.056				0.057
Married	5530 (57.4)	4919 (57.3)	611 (57.9)		1187 (58.5)	600 (59.2)	587 (57.9)	
Divorced	1179 (12.2)	1052 (12.3)	127 (12.0)		253 (12.5)	130 (12.8)	123 (12.1)	
Single	1245 (12.9)	1122 (13.1)	123 (11.6)		225 (11.1)	105 (10.4)	120 (11.8)	
Widowed	1161 (12.0)	1032 (12.0)	129 (12.2)		243 (12.0)	122 (12.0)	121 (11.9)	
Others	526 (5.5)	460 (5.4)	66 (6.2)		120 (5.9)	57 (5.6)	63 (6.2)	
Household income, *n* (%)				0.332				0.047
<50 k	423 (4.4)	397 (4.6)	26 (2.5)		46 (2.3)	20 (2.0)	26 (2.6)	
50–<75 k	2548 (26.4)	2369 (27.6)	179 (17.0)		362 (17.9)	185 (18.2)	177 (17.5)	
75–<100 k	4011 (41.6)	3562 (41.5)	449 (42.5)		863 (42.6)	427 (42.1)	436 (43.0)	
>100 k	2659 (27.6)	2257 (26.3)	402 (38.1)		757 (37.3)	382 (37.7)	375 (37.0)	
Tumor-related characteristics								
Tumor size, mm	14.3 ± 4.1	14.5 ± 4.0	13.1 ± 4.3	0.334	13.2 ± 4.3	13.2 ± 4.4	13.2 ± 4.2	0.009
Tumor laterality, *n* (%)				0.277				0.087
Left	3760 (39.0)	3220 (37.5)	540 (51.1)		994 (49.0)	475 (46.8)	519 (51.2)	
Right	5881 (61.0)	5365 (62.5)	516 (48.9)		1034 (51.0)	539 (53.2)	495 (48.8)	
Tumor site, *n* (%)				0.331				0.057
Lower	3253 (33.7)	2820 (32.8)	433 (41.0)		847 (41.8)	436 (43.0)	411 (40.5)	
Middle	609 (6.3)	598 (7.0)	11 (1.0)		19 (0.9)	8 (0.8)	11 (1.1)	
Upper	5779 (59.9)	5167 (60.2)	612 (58.0)		1162 (57.3)	570 (56.2)	592 (58.4)	
Differentiation grade, *n* (%)				0.110				0.053
Poorly	1458 (15.1)	1312 (15.3)	146 (13.8)		268 (13.2)	126 (12.4)	142 (14.0)	
Moderately	7239 (75.1)	6464 (75.3)	775 (73.4)		1522 (75.0)	772 (76.1)	750 (74.0)	
Well	944 (9.8)	809 (9.4)	135 (12.8)		238 (11.7)	116 (11.4)	122 (12.0)	
T stage, *n* (%)				0.264				0.007
IA1	1841 (19.1)	1535 (17.9)	306 (29.0)		569 (28.1)	283 (27.9)	286 (28.2)	
IA2	7800 (80.9)	7050 (82.1)	750 (71.0)		1459 (71.9)	731 (72.1)	728 (71.8)	
Treatment-related variables								
No. stations of LNs resected				0.394				0.042
0	310 (3.2)	204 (2.4)	106 (10.0)		163 (8.0)	85 (8.4)	78 (7.7)	
1–3	1219 (12.6)	1023 (11.9)	196 (18.6)		356 (17.6)	171 (16.9)	185 (18.2)	
≥4	8112 (84.1)	7358 (85.7)	754 (71.4)		1509 (74.4)	758 (74.8)	751 (74.1)	
No. of LNs resected	9.0 [5.0, 14.0]	9.0 [5.0, 15.0]	6.0 [3.0, 12.0]	0.368	7.0 [3.0, 12.0]	7.0 [4.0, 12.0]	7.0 [3.0, 12.0]	0.024
Adjuvant chemotherapy, *n* (%)	111 (1.2)	106 (1.2)	5 (0.5)	0.083	9 (0.4)	4 (0.4)	5 (0.5)	0.015
Adjuvant radiation, *n* (%)	58 (0.6)	46 (0.5)	12 (1.1)	0.066	15 (0.7)	6 (0.6)	9 (0.9)	0.035
Adjuvant systemic therapy, *n* (%)	118 (1.2)	112 (1.3)	6 (0.6)	0.077	10 (0.5)	4 (0.4)	6 (0.6)	0.028

Data were expressed as mean ± SD, median [25th percentile, 75th percentile] or as frequencies (percentages). Abbreviations: SMD, standardized mean difference; SD, standard deviation; LN, lymph node; T, tumor.

**Table 2:** Survival analyses comparing lobectomy vs. segmentectomy using Cox proportional hazards regression models.

			Crude Model		Model 1		Model 2	
Exposure	Total, *N*	Events (Incidence Rate ^a^)	HR [95% CI]	*p* Value	HR [95% CI]	*p* Value	HR [95% CI]	*p* Value
All-cause mortality								
Lobectomy	1014	158 (35.1)	Ref		Ref		Ref	
Segmentectomy	1014	176 (39.9)	1.14 [0.92, 1.41]	0.233	1.11 [0.89, 1.38]	0.345	1.07 [0.86, 1.34]	0.533
Cancer-specific mortality								
Lobectomy	1014	66 (14.7)	Ref		Ref		Ref	
Segmentectomy	1014	80 (18.1)	1.23 [0.89, 1.71]	0.210	1.20 [0.87, 1.66]	0.276	1.18 [0.84, 1.64]	0.344

^a^ incidence rate: per 1000 person-years. Abbreviations: CI, confidence interval; HR, hazard ratio; Ref, reference; LN, lymph node. Model 1: adjusted for age, sex, and race/ethnicity. Model 2: Model 1+ further adjusted for year at diagnosis, marital status, household income, tumor size, laterality, tumor site, No. stations of LNs resected, No. of LNs resected, adjuvant chemotherapy, adjuvant radiation, adjuvant systemic therapy, and differentiation grade.

**Table 3:** Survival analyses comparing lobectomy vs. segmentectomy stratified by differentiation grade using Cox proportional hazards regression models.

				Crude Model		Model 1		Model 2	
Differentiation Grade	Exposure	Total, *N*	Events (Incidence Rate ^a^)	HR [95% CI]	*p* Value	HR [95% CI]	*p* Value	HR [95% CI]	*p* Value
All-cause mortality									
Poorly	Lobectomy	126	19 (32.5)	Ref		Ref		Ref	
	Segmentectomy	142	26 (36.0)	1.07 [0.59, 1.93]	0.823	1.04 [0.57, 1.88]	0.898	0.84 [0.46, 1.54]	0.571
Moderately	Lobectomy	772	123 (36.3)	Ref		Ref		Ref	
	Segmentectomy	750	138 (44.8)	1.26 [0.99, 1.61]	0.061	1.20 [0.94, 1.54]	0.134	1.18 [0.93, 1.52]	0.178
Well	Lobectomy	116	16 (30.6)	Ref		Ref		Ref	
	Segmentectomy	122	12 (19.8)	0.63 [0.30, 1.33]	0.225	0.69 [0.33, 1.46]	0.336	0.60 [0.28, 1.28]	0.186
Cancer-specific mortality									
Poorly	Lobectomy	126	7 (12.0)	Ref		Ref		Ref	
	Segmentectomy	142	10 (13.9)	1.16 [0.44, 3.04]	0.769	1.12 [0.43, 2.95]	0.815	0.91 [0.34, 2.47]	0.855
Moderately	Lobectomy	772	54 (15.9)	Ref		Ref		Ref	
	Segmentectomy	750	68 (22.1)	1.38 [0.97, 1.97]	0.077	1.33 [0.93, 1.90]	0.118	1.31 [0.91, 1.89]	0.144
Well ^†^	Lobectomy	116	5 (9.6)	Ref		Ref		Ref	
	Segmentectomy	122	2 (3.3)	0.34 [0.07, 1.76] ^†^	0.198	0.36 [0.07, 1.84] ^†^	0.218	0.28 [0.05, 1.46] ^†^	0.130

^a^ incidence rate: per 1000 person-years. ^†^ Estimates based on sparse events (≤5 events) and should be interpreted with caution due to substantial imprecision. Abbreviations: CI, confidence interval; HR, hazard ratio; Ref, reference; LN, lymph node. Model 1: adjusted for age, sex, and race/ethnicity. Model 2: Model 1+ further adjusted for year at diagnosis, marital status, household income, tumor size, laterality, tumor site, No. stations of LNs resected, No. of LNs resected, adjuvant chemotherapy, adjuvant radiation, adjuvant systemic therapy, and differentiation grade.

## Data Availability

The data that support the findings of this study are publicly available from the Surveillance, Epidemiology, and End Results (SEER) database (https://seer.cancer.gov/, accessed on 31 December 2025). The analytical codes used in this study are available from the corresponding authors upon reasonable request.

## Supplementary Materials

The following supporting information can be downloaded at: https://www.mdpi.com/article/10.3390/cancers18081202/s1, Figure S1: Flow chart of patient selection; Table S1: Baseline characteristics of patients stratified by all-cause mortality before and after 1:1 propensity score matching; Table S2: Schoenfeld residual tests for assessing the proportional hazards assumption in Cox regression analyses comparing lobectomy versus segmentectomy in Model 2; Table S3: Survival analyses comparing lobectomy vs. segmentectomy using the Fine–Gray Cox regression model; Table S4: Survival analyses comparing lobectomy vs. segmentectomy using IPTW-Cox proportional hazards regression models; Table S5: Survival analyses comparing lobectomy vs. segmentectomy using new 1:1 propensity score matching parameters.

## Abbreviations

The following abbreviations are used in this manuscript:

LUAD	Lung adenocarcinoma
NSCLC	Non-small cell lung cancer
JCOG	Japan clinical oncology group
CALGB	Cancer and leukemia group B
WJOG	West Japan oncology group
SEER	Surveillance, Epidemiology, and End Results
SD	Standard deviation
OS	Overall survival
LCSS	Lung cancer-specific survival
HRs	Hazard ratios
TNM	Tumor node metastasis
CIs	Confidence intervals
PS	Propensity score
SMD	Standardized mean difference
IPTW	Inverse probability of treatment weighting
ICD-O-3	International classification of diseases for oncology, third edition
IRB	Institutional review board
IASLC	International association for the study of lung cancer
VATS	Video-assisted thoracoscopic surgery
