# Supplementary material for: Segmentectomy Versus Lobectomy in Patients with Stage IA Lung Adenocarcinoma: Long-Term Survival in a Propensity Score-Matched Cohort
Source: Cancers (Basel). 2026 Apr 9;18(8):1202. doi: 10.3390/cancers18081202 (PMC13114701; doi:10.3390/cancers18081202)
Supplement: Supplementary file 1 [file cancers-18-01202-s001.zip › cancers-4204183-supplementary.pdf]

Table S1. Baseline characteristics of patients stratified by all-cause mortality before and after 1:1 propensity score matching.

Characteristics	Before propensity score matching				After propensity score matching			
		All-cause mortality				All-cause mortality		
	Total (n=9,641)	No (n=7,677)	Yes (n=1,964)	SMD	Total (n=2,028)	No (n=1,694)	Yes (n=334)	SMD[a]
Sociodemographic factors								
Age, years	66.3±9.1	65.6±8.9	69.0±9.5	0.378	67.8±8.9	67.1±8.6	70.9±9.4	0.419
Female	6,270 (65.0)	5,176 (67.4)	1,094 (55.7)	0.243	1,377 (67.9)	1,178 (69.5)	199 (59.6)	0.209
Race/ethnicity, n (%)				0.223				0.313
White	7,773 (80.6)	6,091 (79.3)	1,682 (85.6)		1,657 (81.7)	1,357 (80.1)	300 (89.8)	
Black	799 (8.3)	635 (8.3)	164 (8.4)		147 (7.2)	127 (7.5)	20 (6.0)	
Others	1,069 (11.1)	951 (12.4)	118 (6.0)		224 (11.0)	210 (12.4)	14 (4.2)	
Year at diagnosis, n (%)				1.156				1.349
2008–2010	1,345 (14.0)	654 (8.5)	691 (35.2)		183 (9.0)	91 (5.4)	92 (27.5)	
2011–2015	2,630 (27.3)	1,792 (23.3)	838 (42.7)		410 (20.2)	262 (15.5)	148 (44.3)	
2016–2020	4,067 (42.2)	3,649 (47.5)	418 (21.3)		902 (44.5)	812 (47.9)	90 (26.9)	
2021–2022	1,599 (16.6)	1,582 (20.6)	17 (0.9)		533 (26.3)	529 (31.2)	4 (1.2)	
Marital status, n (%)				0.256				0.347
Married	5,530 (57.4)	4,561 (59.4)	969 (49.3)		1,187 (58.5)	1,031 (60.9)	156 (46.7)	
Divorced	1,179 (12.2)	901 (11.7)	278 (14.2)		253 (12.5)	203 (12.0)	50 (15.0)	
Single	1,245 (12.9)	978 (12.7)	267 (13.6)		225 (11.1)	178 (10.5)	47 (14.1)	
Widowed	1,161 (12.0)	807 (10.5)	354 (18.0)		243 (12.0)	177 (10.4)	66 (19.8)	
Others	526 (5.5)	430 (5.6)	96 (4.9)		120 (5.9)	105 (6.2)	15 (4.5)	
Household income, n (%)				0.360				0.348
<50k	423 (4.4)	269 (3.5)	154 (7.8)		46 (2.3)	26 (1.5)	20 (6.0)	
50–<75k	2,548 (26.4)	1,857 (24.2)	691 (35.2)		362 (17.9)	277 (16.4)	85 (25.4)	
75–<100k	4,011 (41.6)	3,267 (42.6)	744 (37.9)		863 (42.6)	738 (43.6)	125 (37.4)	
>100k	2,659 (27.6)	2,284 (29.8)	375 (19.1)		757 (37.3)	653 (38.5)	104 (31.1)	
Tumor-related characteristics								
Tumor size, mm	14.3±4.1	14.2±4.1	15.1±3.9	0.239	13.2±4.3	13.0±4.3	14.4±4.2	0.332
Tumor laterality, n (%)				0.007				0.067
Left	3,760 (39.0)	2,999 (39.1)	761 (38.7)		994 (49.0)	821 (48.5)	173 (51.8)	
Right	5,881 (61.0)	4,678 (60.9)	1,203 (61.3)		1,034 (51.0)	873 (51.5)	161 (48.2)	
Tumor site, n (%)				0.055				0.095
Lower	3,253 (33.7)	2,630 (34.3)	623 (31.7)		847 (41.8)	712 (42.0)	135 (40.4)	
Middle	609 (6.3)	486 (6.3)	123 (6.3)		19 (0.9)	13 (0.8)	6 (1.8)	
Upper	5,779 (59.9)	4,561 (59.4)	1,218 (62.0)		1,162 (57.3)	969 (57.2)	193 (57.8)	
Differentiation grade, n (%)				0.111				0.132
Poorly	1,458 (15.1)	1,203 (15.7)	255 (13.0)		268 (13.2)	223 (13.2)	45 (13.5)	
Moderately	7,239 (75.1)	5,691 (74.1)	1,548 (78.8)		1,522 (75.0)	1,261 (74.4)	261 (78.1)	
Well	944 (9.8)	783 (10.2)	161 (8.2)		238 (11.7)	210 (12.4)	28 (8.4)	
T stage, n (%)				0.151				0.197
IA1	1,841 (19.1)	1,555 (20.3)	286 (14.6)		569 (28.1)	499 (29.5)	70 (21.0)	
IA2	7,800 (80.9)	6,122 (79.7)	1,678 (85.4)		1,459 (71.9)	1,195 (70.5)	264 (79.0)	
Treatment-related variables								
Surgical modality, n (%)				0.065				0.065
Lobectomy	8,585 (89.0)	6,805 (88.6)	1,780 (90.6)		1,014 (50.0)	856 (50.5)	158 (47.3)	
Segmentectomy	1,056 (11.0)	872 (11.4)	184 (9.4)		1,014 (50.0)	838 (49.5)	176 (52.7)	
No. stations of LNs resected				0.302				0.572
0	310 (3.2)	201 (2.6)	109 (5.5)		163 (8.0)	100 (5.9)	63 (18.9)	
1–3	1,219 (12.6)	833 (10.9)	386 (19.7)		356 (17.6)	263 (15.5)	93 (27.8)	
≥4	8,112 (84.1)	6,643 (86.5)	1,469 (74.8)		1,509 (74.4)	1,331 (78.6)	178 (53.3)	
No. of LNs resected	9.0 [5.0, 14.0]	9.0 [5.0, 15.0]	7.0 [3.0, 12.0]	0.256	7.0 [3.0, 12.0]	8.0 [4.0, 13.0]	4.0 [1.0, 9.0]	0.569
Adjuvant chemotherapy, n (%)	111 (1.2)	66 (0.9)	45 (2.3)	0.115	9 (0.4)	6 (0.4)	3 (0.9)	0.069
Adjuvant radiation, n (%)	58 (0.6)	22 (0.3)	36 (1.8)	0.151	15 (0.7)	6 (0.4)	9 (2.7)	0.192
Adjuvant systemic therapy, n (%)	118 (1.2)	72 (0.9)	46 (2.3)	0.111	10 (0.5)	7 (0.4)	3 (0.9)	0.060

Data were expressed as mean±SD, median [25th percentile, 75th percentile] or as frequencies (percentages).

[a]SMDs >0.1 in this table do not indicate inadequate matching, as the stratification is based on outcome status rather than treatment groups.

Abbreviations: SMD, standardized mean difference; SD, standard deviation; LN, lymph node; T, tumor.

Table S2. Schoenfeld residual tests for assessing the proportional hazards assumption in Cox regression analyses comparing lobectomy versus segmentectomy in Model 2.

Exposure variable	Chisq statistics	Degrees of freedom	P-value
All-cause mortality			
Lobectomy vs. Segmentectomy [a]	2.256	1	0.133
(Lobectomy vs. Segmentectomy): differentiation grade[b]	3.513	3	0.319
Cancer-specific mortality			
Lobectomy vs. Segmentectomy [a]	4.117	1	0.834
(Lobectomy vs. Segmentectomy): differentiation grade[b]	5.018	3	0.170

[a]Corresponding to Model 2 in Table 2.
[b]Corresponding to Model 2 in Table 3.
Model 2: adjusted for age, sex, race/ethnicity, year at diagnosis, marital status, household income, tumor size, laterality, tumor site, No. stations of LNs resected, No. of LNs resected, adjuvant chemotherapy, adjuvant radiation, adjuvant systemic therapy, and differentiation grade.

Table S3. Survival analyses comparing lobectomy vs segmentectomy using the Fine-Gray Cox regression model.

			Crude Model		Model 1		Model 2	
Exposure	Total, N	Events (incidence rate[a])	HR [95% CI]	*P* value	HR [95% CI]	*P* value	HR [95% CI]	*P* value
Cancer-specific mortality								
Lobectomy	1,014	66 (14.7)	Ref		Ref		Ref	
Segmentectomy	1,014	80 (18.1)	1.23 [0.89, 1.70]	0.210	1.21 [0.88, 1.68]	0.240	1.15 [0.82, 1.62]	0.410

[a]incidence rate: per 1,000 person-years.
Abbreviations: CI, confidence interval; HR, hazard ratio; Ref, reference; LN, lymph node.
Model 1: adjusted for age, sex and race/ethnicity.
Model 2: Model 1+ further adjusted for year at diagnosis, marital status, household income, tumor size, laterality, tumor site, No. stations of LNs resected, No. of LNs resected, adjuvant chemotherapy, adjuvant radiation, adjuvant systemic therapy, and differentiation grade.

Table S4. Survival analyses comparing lobectomy vs segmentectomy using IPTW-Cox proportional hazards regression models.

				Crude Model		Model 1		Model 2	
Exposure	Outcome	Total, N	Events (incidence rate[a])	HR [95% CI]	*P* value	HR [95% CI]	*P* value	HR [95% CI]	*P* value
All-cause mortality									
Lobectomy	395	5,553	395 (12.9)	Ref		Ref		Ref	
Segmentectomy	47	717	47 (15.0)	1.06 [0.87, 1.29]	0.574	1.04 [0.85, 1.26]	0.705	1.14 [0.94, 1.39]	0.184
Cancer-specific mortality									
Lobectomy	982	5,553	982 (32.2)	Ref		Ref		Ref	
Segmentectomy	112	717	112 (35.7)	1.07 [0.79, 1.44]	0.673	1.06 [0.79, 1.43]	0.702	1.20 [0.91, 1.59]	0.190

[a]incidence rate: per 1,000 person-years.

Abbreviations: IPTW, inverse probability of treatment weighting; CI, confidence interval; HR, hazard ratio; Ref, reference; LN, lymph node.
Model 1: adjusted for age, sex, and race/ethnicity.
Model 2: Model 1 + further adjusted for year at diagnosis, marital status, household income, tumor size, laterality, tumor site, No. stations of LNs resected, No. of LNs resected, adjuvant chemotherapy, adjuvant radiation, adjuvant systemic therapy, and differentiation grade.

Table S5. Survival analyses comparing lobectomy vs segmentectomy using new 1:1 propensity score matching parameters.

			Crude Model		Model 1		Model 2	
Exposure	Total, N	Events (incidence rate[a])	HR [95% CI]	*P* value	HR [95% CI]	*P* value	HR [95% CI]	*P* value
All-cause mortality								
Lobectomy	1,014	154 (35.0)	Ref		Ref		Ref	
Segmentectomy	1,014	170 (39.5)	1.13 [0.91, 1.41]	0.264	1.12 [0.90, 1.40]	0.299	1.08 [0.87, 1.36]	0.482
Cancer-specific mortality								
Lobectomy	1,014	64 (14.5)	Ref		Ref		Ref	
Segmentectomy	1,014	78 (18.1)	1.24 [0.89, 1.73]	0.199	1.21 [0.87, 1.68]	0.266	1.20 [0.86, 1.69]	0.287

[a]incidence rate: per 1,000 person-years.
Abbreviations: CI, confidence interval; HR, hazard ratio; Ref, reference; LN, lymph node.
Model 1: adjusted for age, sex, and race/ethnicity.
Model 2: Model 1+ further adjusted for year at diagnosis, marital status, household income, tumor size, laterality, tumor site, No. stations of LNs resected, No. of LNs resected, adjuvant chemotherapy, adjuvant radiation, adjuvant systemic therapy, and differentiation grade.

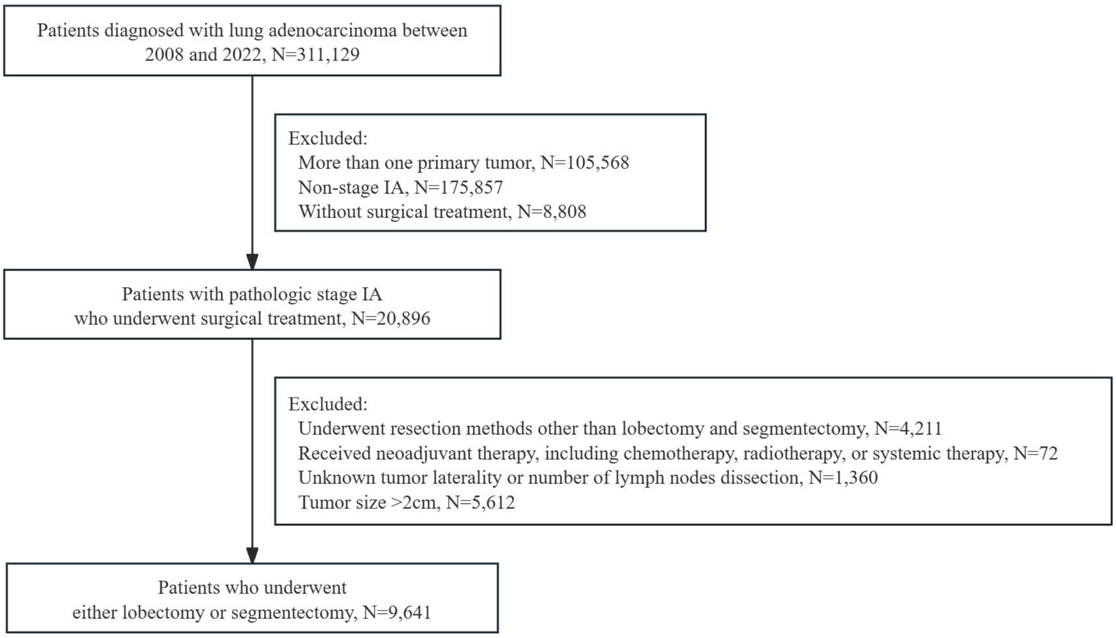

Figure S1. Flow chart of patient selection.
